# ID 289 - O Brasil está Acompanhando as Tendências Mundiais para Tratamentos de Alto Custo? O Exemplo da Doença de Pompe

**DOI:** 10.5327/2237-9622.2025.v34s1.289

**Published:** 2025-11-25

**Authors:** Bruna Bento dos Santos, Cecília de Oliveira Carvalho Faria, Herica Nubia Cardoso Cirilo, Alícia Dorneles Dornelles, Haliton Alves de Oliveira, Ida Vanessa D. Schwartz

**Keywords:** doenças raras, doença de Pompe, economia e organizações de saúde

## Abstract

**Introdução:**

A maioria das doenças raras não possui tratamento específico, e os que estão disponíveis são geralmente de alto custo. O Ministério da Saúde define como raras as doenças que afetam até 65:100.000 indivíduos. A doença de Pompe (DP), também conhecida como glicogenose tipo II ou deficiência de maltase ácida, é um exemplo de doença genética rara com tratamento específico de alto custo. A escolha da DP como modelo neste estudo reflete suas características únicas, que exemplificam os desafios e avanços no acesso a tratamentos de alto custo para doenças raras no Brasil. Este estudo busca caracterizar o acesso a medicamentos de alto custo para doenças raras no Brasil, utilizando a DP como exemplo, e comparar o modelo brasileiro com as tendências globais no tratamento da DP.

**Método:**

Revisão documental abrangente e pesquisa no Sistema de Informação Ambulatorial do Sistema Único de Saúde (SIA/SUS) para a dispensação de alfa-alglicosidase (janeiro/2020 a maio/2024). Os dados de dispensação foram comparados às estimativas de impacto orçamentário para a inclusão da tecnologia no SUS.

**Resultados:**

A alfa-alglicosidase e a alfa-avalglucosidase possuem registro sanitário ativo no Brasil (ambas da mesma fabricante), mas apenas a alfa-alglicosidase está disponível na rede pública, e exclusivamente para pacientes com a DP de início infantil. Houve um intervalo de 12 anos entre o registro sanitário da alfa-alglucosidase e sua avaliação para inclusão no SUS. As estimativas da demanda de frascos de alfa-alglucosidase pela Conitec parecem superestimadas, de acordo com os dados de dispensação do medicamento no SUS. Em comparação com as tendências internacionais, um ponto de convergência entre o Brasil e a maioria dos países avaliados é a recomendação da alfa-alglicosidase exclusivamente para a forma infantil. No entanto, o Brasil ainda se diferencia por não ter avaliado a incorporação da alfa-avalglucosidase no SUS e pela ausência de registro sanitário para a combinação de alfa-cipaglucosidase com miglustate.

**Conclusão:**

O Brasil está parcialmente alinhado com as tendências mundiais no tratamento da DP, mas há diferenças importantes. O País tem avançado no acesso a medicamentos de alto custo por meio de estratégias como a criação do Componente Especializado da Assistência Farmacêutica (Ceaf), que centraliza a compra de medicamentos de alto impacto financeiro, e a priorização de Protocolos Clínicos e Diretrizes Terapêuticas (PCDT) para doenças raras, como a DP. Além disso, a inclusão de medicamentos para doenças raras na Política de Parcerias para o Desenvolvimento Produtivo mostra um esforço crescente para fortalecer a produção local e reduzir a dependência externa. No entanto, o Brasil ainda enfrenta lacunas significativas. A demora de 12 anos entre o registro sanitário e a avaliação para incorporação da alfa-alglucosidase no SUS, apesar da alta demanda judicial, ilustra um importante gargalo. Além disso, faltam políticas robustas para o desenvolvimento e a comercialização de medicamentos órfãos, e o País enfrenta desafios na coleta de dados epidemiológicos sobre doenças raras e na implementação de acordos de compartilhamento de risco. Essas lacunas precisam ser abordadas para garantir que pacientes com doenças raras tenham acesso oportuno a tratamentos seguros e eficazes no SUS.

